# The Impact of Culturing the Organ Preservation Fluid on Solid Organ Transplantation: A Prospective Multicenter Cohort Study

**DOI:** 10.1093/ofid/ofz180

**Published:** 2019-04-26

**Authors:** I Oriol, N Sabe, J Càmara, D Berbel, M A Ballesteros, R Escudero, F Lopez-Medrano, L Linares, O Len, J T Silva, E Oliver, L Soldevila, S Pérez-Recio, L L Guillem, D Camprubí, L LLadó, A Manonelles, J González-Costello, M A Domínguez, M C Fariñas, N Lavid, C González-Rico, L Garcia-Cuello, F Arnaiz de las Revillas, J Fortun, J M Aguado, C Jimenez-Romero, M Bodro, M Almela, D Paredes, A Moreno, C Pérez-Cameo, A Muñoz-Sanz, G Blanco-Fernández, J A Cabo-González, J L García-López, E Nuño, J Carratalà

**Affiliations:** 1 Infectious Disease Department, Hospital Universitari de Bellvitge – IDIBELL; L’Hospitalet de Llobregat, Barcelona, Spain; 2 Spanish Network for Research in Infectious Diseases (REIPI); 3 Clinical Science Department, Faculty of Medicine, University of Barcelona, Barcelona; 4 Microbiology Department, Hospital Universitari de Bellvitge-Universitat de Barcelona-IDIBELL, L’Hospitalet de Llobregat, Spain; 5 CIBER de Enfermedades Respiratorias (CIBERes), Madrid, Spain; 6 Intensive Care Unit, Marqués de Valdecilla Hospital, University of Cantabria, IDIVAL, Santander, Spain; 7 Infectious Diseases Department, Hospital Universitario Ramón y Cajal, Madrid, Spain. IRYCIS; 8 Unit of Infectious Diseases, Hospital Universitario “12 de Octubre”, Instituto de Investigación Sanitaria Hospital “12 de Octubre” (imas12), Madrid, Spain; 9 School of Medicine, Universidad Complutense, Madrid, Spain; 10 Infectious Diseases Department, Hospital Clínic-IDIBAPS, Barcelona, Spain; 11 Infectious Diseases Department, Hospital Universitari Vall d’Hebron, Barcelona, Spain; 12 Universitat Autònoma de Barcelona, Barcelona, Spain; 13 Department of Infectious Diseases, Hospital Universitario de Badajoz, Spain; 14 Donor Coordination Unit, Bellvitge University Hospital, Barcelona, Spain; 15 Liver Transplant Unit, Hospital Universitari de Bellvitge, L’Hospitalet de Llobregat (Barcelona), Spain; 16 Department of Nephrology, Hospital Universitari de Bellvitge, L’Hospitalet de Llobregat (Barcelona), Spain; 17 Department of Cardiology, Hospital Universitari de Bellvitge, L’Hospitalet de Llobregat (Barcelona), Spain; 18 Department of Pathology and Experimental Therapeutics, Faculty of Medicine and Health Sciences, University of Barcelona, Barcelona; 19 Infectious Diseases Unit, Marqués de Valdecilla Hospital, University of Cantabria, IDIVAL, Santander, Spain; 20 Donor Coordination Unit, Marqués de Valdecilla Hospital, University of Cantabria, IDIVAL, Santander, Spain; 21 Department of Internal Medicine, Hospital Universitari Vall d’Hebron, Barcelona, Spain; 22 Liver Transplant Unit, Hospital Universitario de Badajoz, Spain; 23 Kidney Transplant Unit, Hospital Universitario de Badajoz, Spain; 24 Donor Coordination Unit, Hospital universitario de Badajoz, Spain

**Keywords:** preemptive antibiotic therapy, preservation fluid, preservation fluid–related infection, solid organ transplantation

## Abstract

**Background:**

We analyzed the prevalence, etiology, and risk factors of culture-positive preservation fluid and their impact on the management of solid organ transplant recipients.

**Methods:**

From July 2015 to March 2017, 622 episodes of adult solid organ transplants at 7 university hospitals in Spain were prospectively included in the study.

**Results:**

The prevalence of culture-positive preservation fluid was 62.5% (389/622). Nevertheless, in only 25.2% (98/389) of the cases were the isolates considered “high risk” for pathogenicity. After applying a multivariate regression analysis, advanced donor age was the main associated factor for having culture-positive preservation fluid for high-risk microorganisms. Preemptive antibiotic therapy was given to 19.8% (77/389) of the cases. The incidence rate of preservation fluid–related infection was 1.3% (5 recipients); none of these patients had received preemptive therapy. Solid organ transplant (SOT) recipients with high-risk culture-positive preservation fluid receiving preemptive antibiotic therapy presented both a lower cumulative incidence of infection and a lower rate of acute rejection and graft loss compared with those who did not have high-risk culture-positive preservation fluid. After adjusting for age, sex, type of transplant, and prior graft rejection, preemptive antibiotic therapy remained a significant protective factor for 90-day infection.

**Conclusions:**

The routine culture of preservation fluid may be considered a tool that provides information about the contamination of the transplanted organ. Preemptive therapy for SOT recipients with high-risk culture-positive preservation fluid may be useful to avoid preservation fluid–related infections and improve the outcomes of infection, graft loss, and graft rejection in transplant patients.

In spite of many advances, early postoperative infections remain a significant cause of morbidity and mortality among solid organ transplant (SOT) recipients [1]. Therefore, the prevention, diagnosis, and management of infections have become one of the main challenges of transplantation.

Early post-transplant infections are usually derived from the donor or recipient or from technical complications of surgery [2]. The organ donated may be contaminated either by an infection from the donor or as a consequence of the manipulation of the organ in the time between extraction and implantation [3–6]. Moreover, due to its biochemical characteristics, the organ preservation fluid (PF) can keep microorganisms alive and also facilitate their growth [7]. This is why some transplant centers now take intra-operative cultures of the PF to detect infection promptly and avoid its transmission to the recipient. However, there are no widely accepted guidelines for the evaluation of PF or for the use of prophylactic antibiotics [8]. Reliable evidence in support of this practice is scarce and is mainly based on retrospective studies and case reports.

The role of culture-positive PF in the management of transplant recipients has not been fully elucidated. In fact, it is not clear whether the organ PF should be cultured during the transplant procedure or whether preemptive antibiotic treatment (PE-T) is required in SOT recipients with culture-positive PF. In a recent meta-analysis, our group found a high incidence of culture-positive PF, and although few SOT recipients with PF cultures are positive for pathogenic microorganisms, they may develop a PF-related infection with high mortality [9]. However, this meta-analysis was based on the few studies available, which were mainly retrospective; indeed, prospective studies of this subject are lacking.

This prospective multicenter study with a large patient population aims to analyze the incidence, etiology, and risk factors of culture-positive PF and their clinical impact in order to indicate areas for improvement in the management of SOT recipients.

## METHODS

### Setting and Study Population

We conducted a prospective multicenter cohort study at 7 tertiary university referral hospitals in Spain with active transplantation programs (kidney, liver, heart, lung, or pancreas). From July 2015 to March 2017, episodes of SOT in adults were included if the patients or legal surrogates provided written informed consent. Episodes in which the PF was not cultured, in which the recipient died/lost the organ donated within the first 24 hours, or in which informed consent was not provided were not included.

The primary study end point was to assess the incidence rate of PF-related infections. Secondary end points included the prevalence of culture-positive PF, the incidence risk ratio of PF-related infection between SOT recipients who received PE-T and those who did not, and the cumulative incidence of bacterial infections at 90 days and other outcomes at 90 days: graft loss, acute graft rejection (AGR), and mortality.

Data regarding the transplant surgery, as well as the baseline characteristics of SOT donors and SOT recipients, were carefully recorded in an electronic database. All patient data were entered anonymously. To reduce measurement errors, a process of data quality evaluation was used.

Clinical follow-up was performed daily during the post-transplant hospital admission period and then periodically at outpatient appointments, with a post-transplant follow-up period of 3 months. There was no formal or institutional consensus regarding PE-T; the choice was left to the discretion of each attending physician. In cases where PE-T was administered, it was started as soon as the PF culture was found to be positive and was adapted to the resistance profile of the microorganism identified. Similarly, SOT patients received perioperative antibacterial prophylaxis, as well as prophylaxis for opportunistic infections, in accordance with the protocol of each center.

The study was approved by the ethics committees of all participating institutions and conformed to the STROBE checklist.

### Definitions

Culture-positive PF was defined as growth of any microorganism in the PF culture. PF cultures in which the following microorganisms grew were considered to be “high risk”: gram-negative bacilli, *Staphylococcus aureus*, β-hemolytic *streptococci*, *Streptococcus pneumoniae*, *enterococci,* any spore-forming anaerobic gram-positive bacteria, *Bacteroides* species, and *Candida* spp. All the other culture-positive PF were classified as “low risk,” including coagulase-negative staphylococci (CNS), *Corynebacterium* spp., and *Streptococcus viridans* group [5]. PE-T was considered as an immediate post-transplant targeted antibiotic or antifungal treatment against the isolates of culture-positive PF without any clinical signs of active infection in the recipient. PF-related infection was defined as documented infection in the recipient by the same microorganism isolated in the PF culture. Infections were defined according to the Centers for Disease Control and Prevention/National Healthcare Safety Network guidelines [10]. Multidrug resistance (MDR) was defined as acquired nonsusceptibility to at least 1 agent in 3 or more antimicrobial categories [11]. AGR was considered to be present when proven by biopsy, and 90-day mortality was defined as death by any cause within the first 90 days after the onset of SOT.

### Microbiological Studies

Grafts were routinely preserved, mainly in 1 of the following PFs: Celsior, Wisconsin, Perfadex, and Custodiol. PF culture was obtained under sterile conditions just before implantation.

PF samples were processed by the BACTEC FX method (Becton-Dickinson Microbiology Systems, Sparks, MD). The inoculated bottles were incubated for 5 days at 35ºC before being discharged. Microbial identification was performed using commercially available panels (MicroScan, Beckman Coulter; Brea, CA; or Vitek, Biomérieux, Marcy-L’Étoile, France; or by matrix-assisted laser desorption ionization [MALDI-TOF], Bruker Daltonik, Bremen, Germany). Antibiotic susceptibility was tested using the microdilution method following EUCAST guidelines [12]. Molecular typing was performed through pulse field gel electrophoresis after restriction with *XbaI* (enterobacteriaceae) or *SmaI* (staphylococci) following the criteria described by Tenover [13]. The screening of MDR phenotypes including methicillin-resistant *S. aureus*, ampicillin-resistant *enterococci*, extended-spectrum β-lactamase production (ESBL), and carbapenemase production was performed in accordance with EUCAST recommendations.

### Statistical Analysis

We estimated the prevalence of culture-positive PF, the incidence rate of PF-related infections, the incidence risk ratio, and the risk difference of PF-related infections between SOT recipients who received PE-T and those who did not, with confidence intervals.

To compare episodes of SOT by the result of the culture-positive PF, we used the chi-square test with continuity correction for categorical variables and the Student *t* test and Mann-Whitney *U* test for continuous variables. Multivariate conditional regression analysis of factors potentially associated with high-risk culture-positive PF was performed, including all statistically significant variables in the univariate analysis, sex and age, and all clinically important variables regardless of whether they were statistically significant. Odds ratio (ORs) and 95% confidence interval (CIs) were calculated.

Cumulative incidence of bacterial infection among SOT recipients, depending on the result of their PF culture and the decision to give PE-T, was estimated in a competing risk model in which death and graft loss were modeled as competing events. Patients included were censored at the time of (a) death, (b) graft loss, or (c) end of study follow-up. We tested for differences between groups using Fine-Gray regression models [14]. Other secondary outcome variables were compared using the Fisher exact test.

All the statistical management was performed using STATA statistical software, release 13.0 (STATA Corp., College Station, TX). All statistical tests were 2-tailed, and the threshold of statistical significance was *P* < .05.

## RESULTS

During the study period, 622 episodes of SOT were prospectively included (kidney 362, liver 166, lung 51, heart 32, and multi-organ 11). Baseline demographic and clinical characteristics of SOT donors and SOT recipients, including the operation-related data, are summarized in Table 1.

**Table 1.** Baseline Demographic and Clinical Characteristics of SOT Donors and SOT Recipients, Including Surgery-Related Data

Characteristics	n = 622
Donor features	
Age, y	61 (49–72)
Male sex	348 (56.0)
Living donors	39 (6.3)
Brain death donors	449 (72.2)
Donors after circulatory death	134 (21.5)
Length of ICU stay, d	2 (1–5)
Need for vasoactive drugs	412 (66.2)
Prior colonization by resistant microorganisms or fungi	6 (1)
Donor infection	112 (18.0)
Respiratory tract infection	85 (13.7)
Urinary tract infection	7 (1.1)
Central nervous system	7 (1.1)
Other	13 (2.1)
Donor positive cultures	87 (14.0)
Surgery-related features	
Red blood cell transfusion	205 (33.0)
Fresh-frozen plasma transfusion	94 (15.1)
Platelet transfusion	93 (15.0)
Cold ischemia time, min	470 (280–1055)
KT	940 (320–1260)
LT	376 (285–480)
HT	195 (151–225)
PT	350 (295–405)
Length of surgery, min	200 (150–335)
KT	155 (135–185)
LT	375 (313–430)
HT	312 (244–413)
PT	300 (250–360)
Length of antibiotic prophylaxis, h	24 (24–48)
Type of PF	
Celsior	356 (57.2)
Wisconsin	116 (18.7)
Perfadex	43 (6.9)
Custodiol	22 (3.5)
Other	85 (13.7)
Recipient features	
Male sex	416 (66.9)
Age	59 (51–66)
Prior colonization by resistant microorganisms or fungi	30 (4.8)

All data are presented as No. (%) or median (interquartile range).

Abbreviations: HT, heart transplant; ICU, intensive care unit; KT, kidney transplant; LT, liver transplant; MT, multi-organ transplant; PF, preservation fluid; PT, lung transplant; SOT, solid organ transplant.

The prevalence of culture-positive PF was 62.5% (389/622). Most of the isolates were considered “low-risk” microorganisms (291/622), and only 15.8% (98/622) were considered “high-risk” pathogens. Only in 3 cases were the isolates considered MDR. The microorganisms isolated are detailed in full in Table 2.

**Table 2.** Microorganisms Isolated in the PF Culture of SOT

Culture-Positive PF (N = 389)		No. (%)
High risk^a^		98 (15.8)
Monomicrobial		71 (11.4)
Gram-positive bacteria	*Staphylococcus aureus*	19 (4.9)
	*Enterococcus faecalis*	7 (1.8)
	*Enterococcus faecium*	2 (0.5)
	*Streptococcus pneumoniae*	1 (0.2)
	*Streptococcus agalactiae*	1 (0.2)
Gram-negative bacilli	*Escherichia coli*	10 (2.6)
	*Enterobacter cloacae*	5 (1.3)
	*Klebsiella* spp.	4 (1.0)
	*Pseudomonas* spp.	3 (0.8)
	*Serratia* spp.	2 (0.5)
	*Haemophilus influenzae*	2 (0.5)
	Other^b^	10 (2.6)
Anaerobes	*Bacteroides* spp.	1 (0.2)
Fungi	*Candida* spp.^c^	4 (1.0)
Polymicrobial (high-risk +/- low-risk isolates)		27 (6.9)
Low risk^d^		291 (46.8)
Monomicrobial		243 (39.1)
Gram-positive bacteria	CNS	225 (57.8)
	Other^e^	15 (3.9)
Anaerobes	Other^f^	3 (0.5)
Polymicrobial (only low-risk microorganisms)		48 (12.3)

Abbreviations: CNS, coagulase-negative staphylococci; CP, culture positive; PF, preservation fluid.

^a^High risk: gram-negative bacilli, *Staphylococcus aureus*, β-hemolytic *Streptococcus* species, *Streptococcus pneumoniae*, *Enterococci, Bacteroides,* any spore-forming anaerobic gram-positive bacteria, and *Candida* spp.

^b^Other gram-negative bacilli: *Citrobacter freundii* (1), *Burkholderia cepacia* (1), *Stenotrophomonas maltophilia* (1), *Cupriavidus gilardii* (1), *Hafnia alvei* (1), *Raultella planticola* (1), *Rothia mucilaginosa* (1), other nonspecified gram-negative bacilli (3).

^c^ *Candida* spp.: *C. glabrata* (2), *C. albicans* (1), *C. tropicalis* (1).

^d^All microorganisms except those classified as high risk.

^e^Other gram-positive bacteria: viridans group streptococci (3), *Bacillus cereus* (2), *Corynebacterium* spp. (3), *Lactobacillus* (1), *Micrococcus* (3), *Aerococcus viridans* (1), other (2).

^f^Anaerobes: *Prevotella* (1), *Bacteroides* (1), *Propionibacterium* (1), *Peptoniphilus harei* (1).

Table 3 summarizes the risk factors for high-risk culture-positive PF. After applying a backward stepwise logistic regression model, advanced donor age (OR, 1.88; 95% CI, 1.16–3.05) was found to be an independent risk factor for culture-positive PF by high-risk microorganisms.

**Table 3.** Univariate and Multivariate Analyses of Factors Associated With High-risk Culture-Positive PF

Variables	High-risk PF^a^ (n = 98, 15.6%)	Low-risk^b^ or Culture-Negative PF (n = 524, 84.2%)	*P* Value	Adjusted OR (95% CI)	*P* Value
Sex of donors (male)	53 (54.1)	295 (56.1)	.656		
Type of donation			.141		
Living donors	10 (10.2)	29 (5.5)			
Brain death donors	73 (74.5)	374 (71.4)			
Donation after circulatory death	15 (15.3)	119 (22.7)			
Type of transplant					
KT	47 (48.0)	315 (60.1)	.025	0.51(0.11–2.51)	.411
LT	33 (33.7)	133 (25.4)	.089	0.86 (0.17–4.27)	.852
HT	1 (1.0)	31 (5.9)	.044	0.15 (0.01–1.86)	.140
PT	15 (15.3)	36 (6.9)	.005	1.77 (0.34–9.31)	.501
MT	2 (2.0)	9 (1.7)	.824		
Donor infection	12 (12.2)	100 (19.2)	.103		
Mean ischemia time	578 (485–672)	678 (603–753)	.259		
Advanced donor age^c^	81 (82.7)	383 (73.1)	.046	1.88 (1.16–3.05)	.010
ICU days of donor	3.1 (2.3–3.9)	3.6 (3.2–3.9)	.317		

All data are presented as No. (%) or median (interquartile range).

Abbreviations: CI, confidence interval; HT, heart transplant; ICU, intensive care unit; KT, kidney transplant; LT, liver transplant; MT, multi-organ transplant; OR, odds ratio; PF, preservation fluid; PT, lung transplant.

^a^High risk: gram-negative bacilli, *Staphylococcus aureus*, β-hemolytic *Streptococcus* species, *Streptococcus pneumoniae*, *Enterococci, Bacteroides,* any spore-forming anaerobic gram-positive bacteria, and *Candida* spp.

^b^All microorganisms except those classified as high risk.

^c^Donor older than 60 years.

The median length of antibiotic prophylaxis (interquartile range [IQR]) was 24 (24–48) hours. The isolated microorganisms were sensitive to the antibiotic prophylaxis administered during transplantation in 74% of high-risk SOT recipients. PE-T covering the PF isolate was given to 19.8% (77/389) of the culture-positive PF cases (51 due to high-risk and 26 due to low-risk microorganisms). The percentage of high-risk cases that received PE-T varied according to the transplanted organ: 13% (2/15) in lung transplant, 54% (18/33) in liver transplant, 60% (28/47) in kidney transplant, and 100% (1/1) in heart transplant. The median length of PE-T (IQR) was 6 (4–12) days. The median duration of PE-T (IQR) was different according to the microorganisms isolated in the PF culture: 5 (4–7) days for CNS isolation, 5 (3–8) days for *S. aureus* isolation, 4 (3–18) days for *Enterococci*, 8 (5–13) days for *Enterobacteriaceae*, 14 (6–17) days for *Pseudomonas* spp., and 13 (13–46) days in those SOT recipients with growth of *Candida* spp. in their PF.

PF-related infection occurred in 5 recipients, representing a cumulative incidence of 1.3% of SOT recipients with culture-positive PF. The median time from transplantation to the onset of PF-related infection was 6 days. The clinical characteristics of PF-related infections are summarized in Table 4. In the case of *Enterobacter cloacae*, PF-related infection clonality was demonstrated between the strains of the PF culture and the biological sample by the molecular epidemiology study.

**Table 4.** Clinical Characteristics of PF-Related Infections

Cases	Type of Transplant	Sex of SOT Recipient	Age of Recipient, y	Days From Transplant to Infection	Microorganism Isolated	Type of Infection	Days of ICU Post-transplant	AGR	Graft Loss	Re-intervention	90-Day Mortality
1	LT	Male	47	5	*E. faecium*	Intra-abdominal infection	6	No	No	Yes	No
2	HT	Male	19	7	*S. epidermidis*	Surgical site infection	14	No	No	Yes	No
3	PT	Female	58	28	*S. aureus*	Respiratory tract infection	11	Yes	No	No	No
4	PT	Male	28	6	*E. cloacae*	Respiratory tract infection	10	Yes	No	No	No
5	PT	Male	64	2	*S. marcescens*	Respiratory tract infection	90	No	No	No	No

Abbreviations: AGR, acute graft rejection; ICU, intensive care unit; HT, heart transplant; LT, liver transplant; PT, lung transplant; SOT, solid organ transplant.

The incidence risk ratio of PF-related infection between treated and untreated SOT recipients could not be calculated because no PF-related infections were detected in the PE-T group. No statistically significant differences were detected when analyzing the difference in risk of PF-related infection between treated and untreated culture-positive PF, nor when analyzing the low-risk group separately. However, the difference in risk of PF-related infection due to high-risk pathogens between patients who received PE-T and those who did not was 8.5% (95% CI, 0.5%–16.5%; *P* = .033).

**Figure 1.**
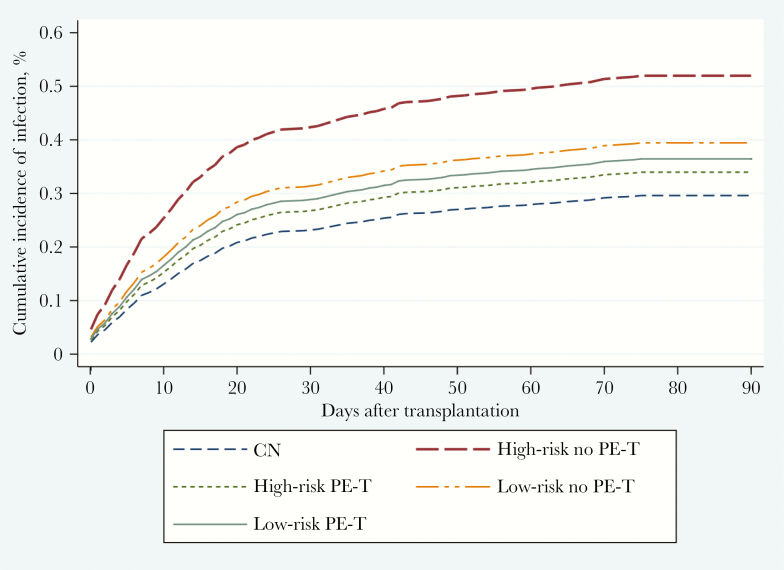
Cumulative incidence of infection on solid organ transplant recipients depending on the result of the preservation fluid culture and the decision to carry out PE-T. Abbreviations: CN, culture-negative preservation fluid; PE-T, preemptive antibiotic therapy.

Figure 1 shows the cumulative incidences of infection in SOT recipients depending on the result of the PF culture and the decision to carry out PE-T.

SOT recipients with culture-positive PF experienced a higher cumulative incidence of infection than the other group (Supplementary Table 1). Analyzing only SOT recipients with culture-positive PF, the 90-day cumulative incidence of infection was lower in the PE-T group. Likewise, among SOT recipients with culture-positive PF high-risk microorganisms, the 90-day cumulative incidence of infection was lower in the PE-T group. No differences between groups were detected in the low-risk group.

After adjusting for sex, age of SOT recipient, type of transplant, and prior episode of AGR, the Fine-Gray analyses revealed that SOT recipients with culture-positive PF by high-risk microorganisms receiving PE-T had a lower cumulative incidence of infection (subhazard ratio, 0.46; 95% CI, 0.24–0.88) (Supplementary Table 2)

There were no significant differences either in the rate of ESBL-producing strains (25.9% vs 16.2%, *P* = .227) or in MDR isolates (7.4% vs 6.9%, *P* = .929) between the infections in SOT recipients who received PE-T and those who did not.

The Supplementary Data (Supplementary Table 3) show the comparison of other outcomes between SOT recipients depending on the result of the PF culture and the decision to carry out PE-T. SOT recipients with culture-positive PF had a higher rate of AGR and mortality than those with culture-negative PF. Among SOT recipients with culture-positive PF, those who received PE-T had a lower risk of AGR. SOT recipients with culture-positive PF high-risk microorganisms who received PE-T experienced a lower frequency of AGR and graft loss than those who did not. No differences in outcomes between groups were detected in the low-risk group. No adjusted analysis was applied due to the small number of events.

## DISCUSSION

This prospective multicenter cohort study is the largest carried out so far to analyze the incidence rate and outcomes of culture-positive PF. We found a high incidence of culture-positive PF, although high-risk microorganisms were isolated in only 15.8% of the cases. Although prior data were mainly derived from retrospective cohorts and showed a wide variability between studies, our results are similar to those of previous prospective studies and support their findings [4, 15].

Advanced donor age was the main associated factor for PF that was culture-positive for high-risk microorganisms. Previous studies have hinted at an association between older donors and PF contamination but have been unable to demonstrate statistical significance in their multivariate analyses. Unlike Cerutti et al., we did not find an association between prolonged ICU stay and fluid contamination [7]. Interestingly, Sotiropoulos et al. analyzed data from 976 SOT donors and concluded that only donor leukocyte count was independently associated with contamination of the PF in SOT [16]. Regrettably, we did not include this variable in our analysis.

In our study, PF-related infections were detected in only 1.3% of all SOT recipients with culture-positive PF, although the rate increased to 8.5% in the case of SOT recipients with high-risk culture-positive PF without PE-T. These rates are consistent with previous reports [17–20]. The high incidence of culture-positive PF and the low rate of PF-related infection are the reasons why some authors do not recommend routine PF culture; they argue that the benefit of treatment is low and that the risk of selecting resistant microorganisms may be increased [15, 21]. Nevertheless, the mortality rate of PF-related infections reported in other studies [7, 22, 23] has encouraged some authors to recommend a short course of PE-T in those SOT recipients with growth of microorganisms in their PF culture [24–26].

We did not detect any PF-related mortality. This conflicting result may be explained, at least in part, by the fact that we did not detect any PF-related infection by *Candida* spp., whose mortality rate (and the rate of graft loss described in case series and cohort studies) is between 50% and 100% of cases [7, 26–28]. The prospective nature of the study may have contributed to the greater diagnostic sensitivity and to the earlier initiation of treatment.

A striking finding of this study was the fact that SOT recipients with culture-positive PF had worse outcomes than those with culture-negative PF. The difference in mortality was at the limit of statistical significance. Our results confirm previous findings by Yansouni et al., who in a retrospective series detected a greater number of infections in SOT recipients with culture-positive PF and a higher mortality rate in liver transplant recipients with culture-positive PF [5]. In contrast, Janny et al. did not detect significant differences between culture-positive and culture-negative PF bacteremia, although this may have been due to the small sample size in their study [27]. Likewise, Chaim et al. detected a higher frequency of AGR among SOT recipients with culture-positive PF than in those with culture-negative PF [22]. An increase in the frequency of AGR is probably seen when immunosuppressive treatment is reduced in order to ​​avoid post-transplant infection. However, at present little is known about the relation of rejection and infection, and studies addressing this issue are lacking.

The reasons why SOT recipients of an organ with culture-positive PF have worse outcomes have not been established. However, the result of the PF culture might be considered as an overall indicator of the quality of the SOT (including the donated organ and the transplant procedure).

Our results show that PE-T only improves the outcomes of infection, graft loss, and AGR in the case of high-risk culture-positive PF. Furthermore, the administration of PE-T in these SOT recipients did not increase the percentage of ESBL isolation and MDR strains in subsequent infections. It should be noted that the median duration of PE-T was less than a week, and the median of transplant antibiotic prophylaxis did not reach 2 days.

Among the strengths of this study are its prospective design, the inclusion of the largest number of SOT episodes described so far, and the fact that the study replicates usual clinical practice. Nevertheless, our research has some limitations that should be noted. We analyzed a heterogeneous group of SOT recipients, who may have had their own specific incidence rates of culture-positive PF and infection. Moreover, length of PE-T was not preestablished. Furthermore, we were unable to perform molecular epidemiology studies in most of the cases that were considered PF-related infections.

In conclusion, the routine culture of the organ preservation fluid may be considered as a tool that provides information about the contamination of the transplanted organ, whether transmitted by the donor or secondary to the transplant procedure. Preemptive antibiotic therapy for SOT recipients with high-risk culture-positive PF may be useful to avoid preservation fluid–related infections and to improve the outcomes of infection, graft loss, and acute graft rejection in transplant patients. Further studies are required to establish the optimal length of PE-T days and long-term outcomes.

## Supplementary Data

Supplementary materials are available at *Open Forum Infectious Diseases* online. Consisting of data provided by the authors to benefit the reader, the posted materials are not copyedited and are the sole responsibility of the authors, so questions or comments should be addressed to the corresponding author.

ofz180_suppl_supplementary_tables_1-3
