# Supplementary material for: The Impact of Culturing the Organ Preservation Fluid on Solid Organ Transplantation: A Prospective Multicenter Cohort Study
Source: Open Forum Infect Dis. 2019 Apr 26;6(6):ofz180. doi: 10.1093/ofid/ofz180 (PMC6546202; doi:10.1093/ofid/ofz180)
Supplement: ofz180_suppl_supplementary_tables_1-3 [file ofz180_suppl_supplementary_tables_1-3.docx]

Table S1: Unadjusted Subdistribution Hazard Ratios (and 95% Confidence Intervals) for 90-days infection by result of the organ PF and decision for carry out PE-T or not.

| **Variables, n (%)** | | **Cumulative incidence of infection at 90 days** | **SHR (95% CI)** | **p-value** |
| --- | --- | --- | --- | --- |
| **All SOT recipients**  **622 (100%)** | **CP**  **389 (62.5%)** | 39.6% (34.7%-44.4%) | 1.41 (1.07-1.85) | 0.014 |
|  | **CN**  **233 (37.5%)** | 30.5% (24.7%-36.4%) |  |  |
| **SOT recipients with Culture positive PF**  **389 (62.5%)** | **PE-T**  **77 (12.4%)** | 35.1% (24.6%-45.7%) | 0.79 (0.53-1.19) | 0.263 |
|  | **No PE-T**  **312 (50.2%)** | 40.7% (35.2%-46.1%) |  |  |
| **“high risk”**  **group^1^**  **98 (15.8%)** | **PE-T**  **51 (8.2%)** | 35.3% (22.6%-48.2%) | 0.57 (0.31-1.03) | 0.062 |
|  | **No PE-T**  **47 (7.6%)** | 51.1% (36.1%-64.2%) |  |  |
| **“low risk”**  **group^2^**  **291 (46.8%)** | **PE-T**  **26 (8.9%)** | 34.6% (17.5%-52.5%) | 0.89 (0.44-1.82) | 0.750 |
|  | **No PE-T**  **265 (91.1%)** | 38.9% (33.0%-44.7%) |  |  |

AGR: acute graft rejection; CI: confidence interval; CN: culture-negative preservation fluid; CP: culture-positive preservation fluid; SHR: Subdistribution Hazard Ratio; PE-T: pre-emptive therapy.

^1^ “high risk”: *Gram-negative bacilli*, *Staphylococcus aureus*, β-haemolytic streptococcus species, *Streptococcus pneumoniae*, *Enterococci, Bacteroides,* any spore-forming anaerobic gram-positive bacteria and *Candida* spp.

^2^ All microorganisms except those classified as “high risk”.

Table S2. Unadjusted and adjusted Subdistribution Hazard ratios of association between “high risk” culture-positive PF and cumulative incidence of bacterial infection.

| **Variables** | **SHR (95% CI)** | **p-value** | **aSHR (95%CI)** | **p-value** |
| --- | --- | --- | --- | --- |
| **Sex** | 0.81 (0.44-1.49) | 0.500 | 0.92 (0.47-1.81) | 0.816 |
| **Age of recipient** | 0.74 (0.40-1.36) | 0.332 | 0.99 (0.96-1.03) | 0.732 |
| **Type of transplant** |  | 0.136 |  |  |
| **LT** | 1.17 (0.86-1.59) | 0.327 | 1.83 (0.93-3.89) | 0.078 |
| **HT** | 1.75 (0.94-3.28) | 0.080 | 3.48e-07  (2.08e-08-5.82e-06) | <0.001 |
| **PT** | 1.43 (0.88-2.31) | 0.150 | 1.07 (0.31-3.72) | 0.910 |
| **MT** | 1.97 (0.88-4.41) | 0.101 | 8.47 (4.12-17.41) | <0.001 |
| **Prior AGR** | 0.42 (0.20-0.87) | 0.020 | 0.16 (0.02-1.14) | 0.067 |
| **Length of antibiotic prophylaxis** | 1.00 (0.99-1.00) | 0.982 |  |  |
| **Advanced donor age^1^** | 1.01 (0.78-1.31) | 0.938 |  |  |
| **PE-T** | 0.57 (0.31-1.03) | 0.062 | 0.46 (0.24-0.88) | 0.019 |

SHR: Subdistribution Hazard Ratio; CI: confidence interval; LT: liver transplant; HT: heart transplant; PT: lung transplant; MT: multiple transplant; AGR: acute graft rejection; PE-T: pre-emptive antibiotic therapy.

^1^Donor older than 60 years.

Table S3.Other outcomes at 90 days among SOT recipients depending on the result of PF culture and the decision to carry out PE-T.

| **Outcomes** | **All series (N=622)** | | | **CP (N=389)** | | | **“high risk” group^1^ (N=98)** | | | **“low risk” group^2^ (N=291)** | | |
| --- | --- | --- | --- | --- | --- | --- | --- | --- | --- | --- | --- | --- |
|  | **CP** | **CN** | **p-value** | **PE-T** | **No**  **PE-T** | **p-value** | **PE-T** | **No**  **PE-T** | **p-value** | **PE-T** | **No**  **PE-T** | **p-value** |
| **AGR** | 46 (11.8%) | 15 (6.4%) | 0.036 | 4 (5.2%) | 42 (13.5%) | 0.048 | 3 (5.9%) | 11 (23.4%) | 0.019 | 1 (3.9%) | 31 (11.7%) | 0.331 |
| **Graft loss** | 17 (4.4%) | 8 (3.4%) | 0.675 | 1 (1.3%) | 16 (5.1%) | 0.213 | 0 | 4 (8.5%) | 0.049 | 1 (3.9%) | 12 (4.5%) | 1 |
| **90-days mortality** | 14 (3.6%) | 2 (0.9%) | 0.038 | 3 (3.9%) | 11 (3.5%) | 0.745 | 1 (2.0%) | 2 (4.3%) | 0.606 | 2 (7.7%) | 9 (3.4%) | 0.257 |

AGR: acute graft rejection; CN: culture-negative preservation fluid; CP: culture-positive preservation fluid; PE-T: pre-emptive therapy.

^1^ “high risk”: *Gram-negative bacilli*, *Staphylococcus aureus*, β-haemolytic streptococcus species, *Streptococcus pneumoniae*, *Enterococci, Bacteroides,* any spore-forming anaerobic gram-positive bacteria and *Candida* spp.

^2^ All microorganisms except those classified as “high risk”.
